# Hydration and Body Composition in Sports Practice: An Editorial

**DOI:** 10.3390/nu15224814

**Published:** 2023-11-17

**Authors:** Francesco Campa

**Affiliations:** Department of Biomedical Sciences, University of Padua, 35131 Padova, Italy; francesco.campa@unipd.it

Assessing hydration status and monitoring body composition represent crucial aspects when discussing the advantages of embracing a healthy lifestyle, given its significant impact on both health and sports performance [1,2]. Over recent years, the realm of sports-related research has expanded and become deeper, providing a solid foundation for the creation of innovative assessment methodologies designed to enhance body composition, overall health, and physical performance [3,4]. The articles featured in this research compilation underscore the intricate relationships among these specific parameters through a combination of longitudinal and cross-sectional experimental designs, as well as systematic literature reviews. Contemporary researchers have made valuable contributions to the field of health improvement and sports performance enhancement by introducing novel measurement techniques for evaluating body composition and devising training strategies to optimize body composition and sports-related achievements [5,6].

Many researchers are currently focusing their efforts on studying the use of supplements in sports, as well as delving into the impact of dehydration on athletic performance [5,7,8]. The exploration of supplements aims to uncover their potential benefits in enhancing various aspects of sports performance, such as muscle recovery, endurance, and strength [9,10]. Simultaneously, the investigation into the effects of dehydration on athletic performance seeks to understand how inadequate hydration can negatively influence an athlete’s stamina, cognitive function, and overall results during physical activities [11,12,13]. These research areas are pivotal in helping athletes and coaches make informed decisions to optimize their training and competitive outcomes. It has been shown that elite female soccer players experience higher sweat rates during matches in comparison to their training sessions [14]. The notable variability in individual sweat rates and sodium concentration in sweat during both training and matches highlights the necessity for personalized hydration recommendations for this group. Additionally, the observed insufficient carbohydrate intake among these athletes during both training and matches warrants a more comprehensive investigation, emphasizing the urgent requirement for enhanced nutritional support from sports nutrition experts in the realm of elite women’s soccer. Research has also demonstrated that even mild isolated dehydration (hypohydration) can result in decreased workload capacity and oxygen uptake among highly motivated recreational athletes [15]. This decline in exercise performance is attributed to a noteworthy reduction in oxygen pulse, suggesting that the cause of this impairment lies in the reduced cardiac output due to a decrease in preload. These findings, which reveal a substantial reduction in exercise capacity with only mild dehydration, point to the potentially pivotal role of dehydration in the heat-induced decrease in exercise performance. Athletes often use various dietary supplements to optimize their body composition while ensuring they meet their nutritional requirements for rigorous training and competition. The majority of open water swimmers, approximately 80%, consume dietary supplements, especially those at a higher competitive level [16]. The prevailing consumption pattern includes sports nutrition products like sports drinks and energy bars, performance-enhancing supplements such as caffeine, and medical supplements like vitamins C and D. These supplements fall within the category supported by substantial scientific evidence. Although the consumption of supplements lacking scientific support for athletic benefits or without relevant research (Group C as defined by AIS) is relatively low, accounting for less than 20%, some swimmers do use them.

The measurement of body fat is a common practice in the world of sports [17]. Accurate assessments of body fat percentage are essential for athletes to monitor their progress, make informed training and nutrition decisions, and fine-tune their performance strategies, ultimately helping them achieve their competitive goals and maintain peak physical condition. Current scientific research is increasingly dedicated to developing predictive formulas tailored to estimate fat mass in specific sports [18]. These formulas serve as valuable tools for athletes and coaches, aiding in the precise assessment of body composition and enabling more targeted training and dietary interventions to optimize performance. A novel formula [19], based on a combination of anthropometric measurements, is now accessible for estimating fat mass in high-level futsal players. This formula is as follows: −0.620 + (0.159 × sum of skinfold thickness measurements at four sites: triceps, abdominal, iliac crest, and front thigh (in millimeters)) + (0.120 × waist girth measurement in centimeters). Field methods like anthropometry and bioimpedance have gained significant popularity in the realm of sports [20]. However, the demand for tailored equations designed specifically for athletes has become increasingly apparent. These equations are essential for accurately assessing body composition in the athletic population and supporting their performance optimization and training regimens [17].

In recent years, there has been a surge in research interest regarding the role of gut microbiota in the context of sports [21,22]. Scientists are increasingly delving into the intricate interactions between the gut microbiome and an athlete’s performance, recovery, and overall health, recognizing the potential impact of this emerging field on optimizing athletic outcomes [23,24]. While the body of literature examining the influence of physical activity on gut microbiota is steadily growing, uncertainties persist regarding the precise effects of physical activity on bacterial flora [25]. It appears that voluntarily performed exercise can help mitigate intestinal inflammation, whereas forced physical activity has shown the potential to exacerbate this condition in animal models. In the realm of human studies, vigorous endurance exercise has been linked to adverse effects on the gut microbiota framework, particularly observed in aerobic activities, possibly due to the positive correlation between cardiorespiratory fitness and microbial diversity. Physical activity can enhance the richness of bacterial communities by promoting the growth of short-chain fatty acid (SCFA)-producing species and facilitating the colonization of beneficial strains such as Akkermansia muciniphila and Veillonella. These changes in gut microbiota composition support intestinal mucosal protection against permeability issues and counteract alterations induced by a high-fat diet. Collectively, physical activity is associated with an increased relative abundance of the Bacteroidetes phylum and a reduction in Firmicutes, with potentially more pronounced and enduring benefits when initiated at a young age. However, the impact on gut microbiota composition appears to gradually diminish when physical activity is discontinued. Furthermore, physical activity shows promise in regulating cognitive conditions such as anxiety and depression, as well as functionality in conditions like Alzheimer’s and Parkinson’s Disease. These effects are linked to alterations in microbial composition and the subsequent production of specific protective molecules, as evidenced in animal models.

In conclusion, there is a compelling need for further studies dedicated to exploring new methods for assessing hydration status and body composition and their influence on physical performance in the context of sports. As our understanding of these factors continues to evolve, comprehensive research will provide athletes, coaches, and sports professionals with the essential insights required to optimize performance, health, and overall well-being in the world of sports.
